# Analysis of the Frequency of Mutations at Diagnostic Oligonucleotide Sites and Their Impact on the Efficiency of PCR for HIV-1

**DOI:** 10.3390/microorganisms11122838

**Published:** 2023-11-22

**Authors:** Elena V. Bogoslovskaya, Galina M. Tsyganova, Anastasiia O. Nosova, German A. Shipulin

**Affiliations:** Federal State Budgetary Institution “Centre for Strategic Planning and Management of Biomedical Health Risks” of the Federal Medical Biological Agency, 119121 Moscow, Russia; tsyganova@cspmz.ru (G.M.T.); anosova@cspfmba.ru (A.O.N.); shipulin@cspmz.ru (G.A.S.)

**Keywords:** HIV-1, real-time PCR, mutations

## Abstract

The development of effective diagnostic kits for HIV-1 remains a pressing concern. We designed diagnostic oligonucleotides for HIV-1 real-time PCR to target the most conserved region of the HIV-1 genome and assessed the mutation frequency at annealing sites. Two databases of nucleotide sequences, Los Alamos and NCBI, were analyzed, revealing that more than 99% of the sequences either lack mutations or contain 1–2 mutations at the binding site of the forward and reverse primers. Additionally, 98.5% of the sequences either lack mutations or contain 1–2 mutations at the binding site of the TaqMan probe. To evaluate the efficiency of primers and the probe in real-time PCR in the case of mutations at their binding sites, we constructed several plasmids containing the most common mutations and, in a model experiment, showed how different mutations affect the efficiency of PCR. Our analysis demonstrated that about 98.5% of HIV-1 strains can be efficiently detected using a single pair of selected primers. For the remaining 1.5% of strains, a more careful selection of the second target is needed.

## 1. Introduction

HIV-1 viral load (VL) is a critical diagnostic marker for prognosis and evaluating the effectiveness of ARV therapy in HIV-infected patients. Commercial kits for the quantitative detection of HIV-1 RNA (VL) have been developed and used throughout the world for many years. The most common method for determining HIV-1 VL is real-time PCR.

The high variability of HIV-1 leads to the regular emergence of new variants of the virus [1], some of which may be ineffectively detected using long-used commercial kits. In these cases, VL is determined incorrectly (or HIV-1 RNA is not detected at all), which leads to inadequate diagnosis and treatment. To ensure accurate diagnosis and treatment, it is extremely important to regularly analyze existing kits for their effectiveness in the context of an ever-changing virus population, as well as to develop new, more effective kits.

When developing new real-time PCR kits, it is important not only to select the most conserved region of the HIV-1 genome, but also to correctly position the primers and probes to minimize the number of mismatches at their binding sites. Due to the extreme variability of the virus, it is not possible to completely avoid mismatches, but you can reduce their impact on PCR efficiency through varying the position of the primer relative to the mismatch.

The objectives of this study included selecting new diagnostic oligonucleotides for the quantitative detection of HIV-1 RNA using real-time PCR. An important consideration when choosing primers for PCR is the sampling of nucleotide sequences used to analyze the conservation of the selected regions of the HIV-1 genome. The presence of international databases of nucleotide sequences greatly simplifies the task of selecting PCR primers, with the Los Alamos database being one of the best freely available resources. However, the completeness of nucleotide sequence databases varies depending on the genotype and often depends on the volume of sequenced samples.

The prevalence of different genotypes varies significantly in different regions of the world. While genotype B is dominant in the USA and Western European countries, subtype C is the prevalent genotype in Africa. In Russia and Eastern European countries, genotype A is more common [2], and in China, predominantly recombinant forms circulate [3]. As a result, the completeness of databases heavily depends on how actively each country sequences samples and uploads sequences to international publicly available databases. That is why there is a certain bias towards HIV-1 subtypes B and C in international databases, with these subtypes being underrepresented in some countries, particularly in Russia. Therefore, it was also important for us to assess how conserved the selected regions of the HIV-1 genome are among Russian isolates. In addition to theoretical analysis, our task was to evaluate the efficiency of PCR using selected oligonucleotides in model experiments.

## 2. Materials and Methods

### 2.1. Nucleotide Sequence Databases and Alignments

Analysis of the selected oligonucleotides was carried out using two databases: NCBI [4] and Los Alamos [5]. The first database was used to generate data on the diversity of genetic variants of HIV-1 isolated on the territory of the Russian Federation; the second database made it possible to obtain information on HIV-1 sequences from all over the world.

All records of HIV-1 nucleotide sequences containing the keyword “Russia” in the “country” field were downloaded from the NCBI database [4]. From the resulting sample of sequences (9031 records), records that did not contain regions of interest in the sequence (primer and probe binding sites) were excluded. Multiple alignment of nucleotide sequences was performed using MAFFT [6]. A separate alignment for each oligonucleotide was created from the resulting sample. The final alignment for the forward and reverse primer regions was 1206 and 1171, respectively, and for the probe region was 1202.

The second sample was formed in a similar way using ready-made sequence alignment (“web alignment”) of the HIV-1 pol gene published in the Los Alamos HIV database [5]. Sequences containing deletions in the studied regions were excluded. The final sample consisted of 6329 sequences. From the resulting sample separate alignments were created for each oligonucleotide, limiting the available sequences to the region of a particular primer or probe.

For convenient presentation of data in each alignment, sequences were clustered using the CD-HIT program [7] (identity threshold—100%) and the resulting clusters were sorted according to the number of representatives included in them in descending order.

### 2.2. DNA Constructs and Site-Directed Mutagenesis

To obtain DNA PCR calibrators, we used the pBH10 plasmid (obtained through the NIH HIV Reagent Program, Division of AIDS, NIAID, NIH: Human Immunodeficiency Virus 1 (HIV-1) BH10 Non-Infectious Molecular Clone (pBH10), ARP-90, contributed by Dr. Beatrice Hahn and Dr. George M. Shaw), which was diluted to the required concentrations using a series of 10-fold dilutions.

To obtain a control DNA construct, a target fragment of the HIV genome region, including the HIV integrase gene 412 bp long, was generated via PCR using specific primers: Int_for4 CCCTACAATCCCCAAAGTCARGGAGT and Int_rev5 CATCACCTGCCATCTGTTTTTCCATARTC and plasmid pBH10 as a DNA template.

The resulting amplicon was ligated into the pGEM vector (Promega, Madison, WI, USA). The ligase mixture was introduced through transformation into *E. coli* cells, strain DH10b. Screening of the obtained clones was carried out using the PCR method. The structure of the target fragment was confirmed using Sanger sequencing.

To obtain DNA constructs with mutations in the binding sites of the primers and probe, the control DNA construct was used, into which mutations were introduced using the QuikChange II Site-Directed Mutagenesis kit (Agilent, Santa Clara, CA, USA) in accordance with the manufacturer’s recommendations. The resulting DNA constructs were verified via Sanger sequencing.

### 2.3. Determination of Concentrations of DNA Constructs

The concentration of DNA constructs was measured using primers and a probe for the region of the ampicillin resistance gene (the bla gene, which is part of all plasmid constructs and encodes the beta-lactamase protein):

Bla1 tacgggagggcttaccatctg

Bla2 ggctggtttattgctgataaatctg

Bla3 R6G-accgcgagacccacgctcacc-BHQ1

Concentration measurements were conducted using the Digital PCR system, which includes the automatic loading of samples into a QX200™ AutoDG™ droplet generator (BioRad, Hercules, CA, USA) and a C1000 Touch Thermal Cycler (Bio-Rad, Hercules, CA, USA). To perform the analysis, we used the ddPCR™ reagent kit for sample preparation with TaqMan QX100/QX200™ probes (Bio-Rad, Hercules, CA, USA). Next, all samples were diluted to the same concentration value, taking into account the previously obtained values. The concentration of the prepared samples was measured in 5 replicates, and the median of the obtained concentrations in copies per milliliter was used for further studies. The concentration in DNA calibrators was determined using the same method.

### 2.4. Real-Time PCR

For real-time PCR, selected primers and a probe in the region of the HIV integrase gene, in-house reagents, and a RotorGene Q device (Qiagen, Hilden, Germany) were used. To evaluate quantitative results, DNA calibrators with DNA concentrations of 10^5^, 10^4^, and 10^3^ copies per ml were used.

### 2.5. Statistics

For all quantitative values, the mean value and standard deviation were calculated. The significance of differences between data groups was assessed using the Mann–Whitney U-test. Differences were considered statistically significant if the *p*-value was <0.001.

## 3. Results

### 3.1. Oligonucleotide Selection for Real-Time PCR

The target for real-time PCR of HIV-1 was chosen in the integrase gene since it is the most conserved region of HIV-1 [8]. When choosing, we took into account the conservation of the oligos binding site, GC composition, melting temperature, and other factors that may affect the efficiency of PCR.

When selecting primers and probes, we used alignments of HIV-1 nucleotide sequences presented in the Los Alamos database, in particular, the HIV Sequence Compendium 2018 [9]. As a result, a pair of primers was selected to amplify a 237 bp fragment of HIV-1 and TaqMan probe for the detection of amplification products (supplementary materials).

The length and, accordingly, the melting temperature of the primers were selected with higher-than-optimal values so that during PCR, 1–2 mutations at the binding sites would not affect the amplification efficiency.

### 3.2. HIV-1 Nucleotide Sequence Analysis

The selected primers and probe were analyzed using two databases: Los Alamos and NCBI. The alignment of all HIV-1 nucleotide sequences from the Los Alamos database resulted in 6328 nucleotide sequences in the forward primer region, 6324 sequences in the reverse primer region, and 6326 sequences bounded by the probe region. The most prevalent genotype was B, representing over 33% of the sequences. Following closely was genotype C, accounting for approximately 17%. The recombinant form 01_AE was the next most common, making up 9.8% of the sequences. These data further confirm the predominance in international databases of HIV sequences distributed in the United States and African countries, where the highest HIV prevalence is observed.

Additionally, the primers and probe were analyzed using the NCBI database limited to the code word “Russia”. The final alignment in the forward and reverse primer regions contained 1206 and 1171 sequences, respectively. Probe region analysis was performed for 1202 sequences. In this sample, the predominant genotype was A (65.8%), and the second most common was genotype B (7.2%). More than 10% of the sample was occupied by circulating recombinant forms (CRFs) with a predominance of CRF63_02A.

#### Estimation of Mutation Frequency at Primer and Probe Binding Sites

We analyzed the frequency of mutations at the binding sites of the primers and probe and also assessed the position of each mutation in the oligonucleotide. The results are presented in tables and the supplementary materials.

**Figure 1:**
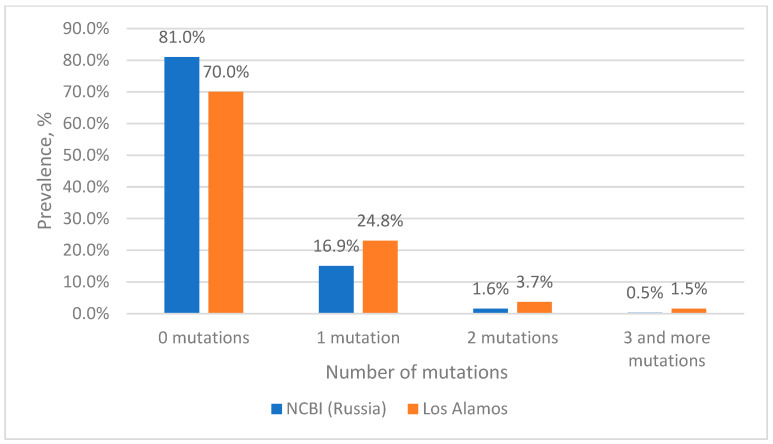
Prevalence of HIV-1 strains with mutations in the probe binding site.

The analysis of nucleotide sequences from the Los Alamos database at the TaqMan probe binding site showed that more than 70% of the sequences are completely complementary to the probe sequence (Figure 1). The remaining 23% of sequences have single mutations at the probe binding site, with clusters 1 to 6 (the most numerous clusters in terms of the number of sequences) accounting for about 21% of all analyzed sequences (supplementary materials, Figure S1). At the same time, two mutations at the probe binding site occur with a frequency of less than 4%, with the most frequent corresponding to clusters 7, 8 and 10 and a total of 1.64%. At the same time, three mutations occur with a frequency of 1.5%, the most frequent ones correspond to clusters 12 and 14, with a total of 0.55%.

When analyzing the nucleotide sequences of Russian isolates from the NCBI database at the binding site of the TaqMan probe, it turned out that more than 81% of the sequences were completely complementary to the probe sequence (Figure 1). Sequences included in clusters 1 to 10 contain single mutations and in total account for 15% (supplementary materials, Figure S2). The two mutations simultaneously occur most frequently in sequences corresponding to cluster 11 (0.42%), which corresponds to cluster 8 from the Los Alamos database (0.63%). With a low frequency (0.17%), two mutations occur simultaneously in the sequences of cluster 17, which corresponds to cluster 10 from the Los Alamos database, with a frequency of occurrence of 0.33%. Three mutations among Russian isolates are extremely rare, accounting for a total of 0.3%.

From the presented data, it is clear that 93–96% of sequences have no substitutions or have one substitution at the probe binding site and theoretically should be effectively detected by the selected probe; however, its effectiveness in the case of single mismatches in the probe binding area requires experimental validation. For this purpose, we subsequently constructed plasmids containing an insertion of a 412 bp fragment of the integrase gene, into which the necessary mutations were introduced. At the same time, two mutations at the probe binding site are also of interest, since they account for about 4% of all sequences. It is worth noting that the most common combination of two mutations among the sequences in the Los Alamos database belongs to cluster 7, which includes sequences of viruses, most of which belong to group O (86%), which are poorly represented in the world and are not found in Russia. At the same time, among Russian isolates from the NCBI database with two mutations, representatives of genotype B (cluster 11) are the most common. Three mutations at the same time are quite rare, no more than 1.5%, so the analysis of such sequences is of rather theoretical interest. However, we also further investigated such variants experimentally.

We designed the forward primer with one degenerate position C/T (fifth nucleotide from the 3′ end) because nucleotide C occurs with a frequency of 6.7% among Russian isolates and with a frequency of 34.8% in the Los Alamos database, and nucleotide T occurs with a frequency of 91.6% among Russian isolates and with a frequency of 63.5% in the Los Alamos database. In further analysis, we did not consider the degenerate position as a mismatch.

**Figure 2:**
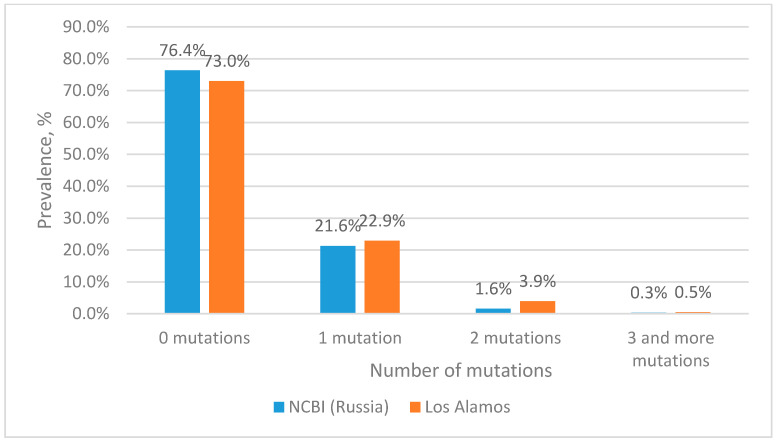
Prevalence of HIV-1 strains with mutations in the forward primer binding site.

The analysis of nucleotide sequences from the Los Alamos database showed that about 73% of the sequences are completely complementary to the forward primer sequence (Figure 2). The remaining 23% consist of sequences containing single mutations, most of which are located closer to the 5′ end, which is known to have little effect on the efficiency of primers. At the same time, two mutations at the site of the forward primer occur with a frequency of less than 4%. Three or more mutations occur with a frequency of less than 0.5%.

When analyzing the nucleotide sequences of Russian isolates from the NCBI database, it turned out that more than 76% of the sequences are fully complementary to the sequence of the forward primer (Figure 2). About 21% of the sequences included in the analysis have one mutation. At the same time, two and three mutations in the forward primer region among Russian isolates occur with a frequency of 1.6% and 0.3%, respectively.

From the data presented in Figure 2, it is clear that more than 99% of the sequences either do not contain mutations or contain 1–2 mutations at the binding site of the forward primer. According to our calculations, the presence of two mutations at the binding site of the selected primers should not affect the efficiency of amplification. For subsequent experimental evaluation we constructed plasmids containing a fragment of the integrase gene into which 3 or more mutations were introduced.

When choosing a reverse primer, we faced the fact that in two positions there was a strong discrepancy in the frequency of occurrence of nucleotides between isolates from the international database and Russian isolates (supplementary materials). Thus, in position 2 from the 5′ end, nucleotide C occurs with a frequency of 84% among Russian isolates and with a frequency of 16% in the international database. A similar situation is observed at position 11 from the 5′ end, in which the nucleotide C occurs with a frequency of 82.8% among Russian isolates and with a frequency of 4.4% in the international database. It was decided to leave nucleotide C at position 2 at the 5′ end, since mutations in this position have virtually no effect on the efficiency of amplification and since this nucleotide is more common among Russian isolates. At position 11 (i.e., almost the middle of the primer) we made a degenerate position C/T. In further analysis, the degenerate position was not considered a mismatch.

**Figure 3:**
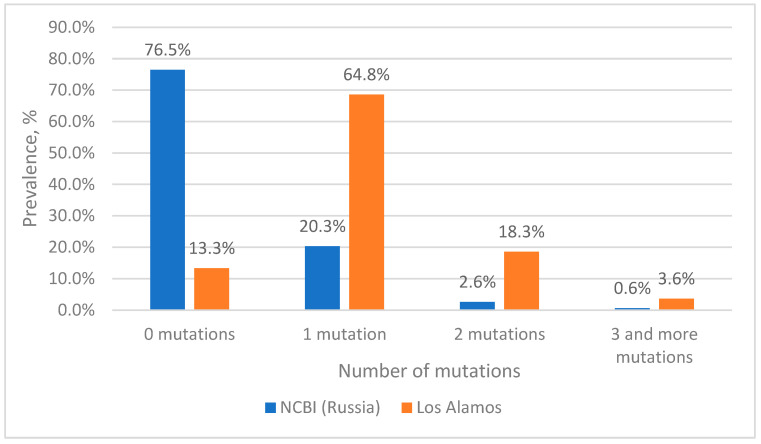
Prevalence of HIV-1 strains with mutations in the reverse primer binding site.

When analyzing nucleotide sequences from the Los Alamos database, it is clear that complete complementarity to the reverse primer is observed in 13% of cases (Figure 3); this is due to the fact that we left the mutation in position 2 from the 5′ end of the primer. Additionally, 68% of the analyzed sequences had a single mutation at the primer binding site, with position 2 from the 5′ end of the primer accounting for 62%.

Simultaneously, two mutations at the site of the reverse primer occur with a frequency of 18%. At the same time, three or more mutations occur with a frequency of 3.6%.

Although a total of three or more mutations occurred quite often (3.6%), each combination occurred no more often than 0.2%. The exception was sequences from cluster 8 (0.65%), in which four mutations occur simultaneously; most of the sequences belong to the group O virus (85%). Therefore, when constructing plasmids containing mutations for model experiments, preference was given to those mutations that could maximally reduce the efficiency of the primer, i.e., were located closer to the 3’ end or there were four and five mutations.

When analyzing the nucleotide sequences of Russian isolates from the NCBI database, it turned out that more than 76% of the sequences are completely complementary to the reverse primer (Figure 3). Among sequences with mutations, the most common are variants with single substitutions—20%. The highest percentage of occurrence is for sequences with adenine in position 2 from the 5′ end of the primer (12.6%). Simultaneously, two mutations are found in 2.6% of sequences, of which the most common variant unites about 1% of sequences (cluster 4). Three or more mutations are rare, accounting for a total of 0.6% of sequences, and, moreover, each specific variant is represented in the NCBI database by a single sequence.

### 3.3. Efficiency of Primers and Probe in Real-Time PCR in Case of Mismatches at Binding Sites

In order to evaluate the efficiency of primers and probes in real-time PCR in the case of mutations at their binding sites, we constructed several plasmids containing the amplification region of the integrase gene fragment, into which the most common mutations were introduced. The resulting DNA plasmids with equal concentration were tested together with the mutation-free plasmid in real-time PCR using the selected primers and probe. In parallel, DNA calibrators were tested in each setup to obtain quantitative values for each sample.

Table 1 presents the constructed plasmids, along with details of the introduced mutations and the frequency of nucleotide sequences with these mutations in two databases.

In the case of the probe, we made the largest number of plasmids, dividing them into three groups. The first group included constructs with the most common single mutations at the probe binding site, the second group included constructs with two mutations, and the third group, three mutations. The third group was of only theoretical interest, since it was obvious that the probe would not work effectively in the case of three mutations.

Since we deliberately chose primers with a higher melting temperature, taking into account the possible presence of 1–2 mutations at the binding site, plasmids were constructed containing 3 or more mutations at the binding sites of the primers (Table 1). The frequency of such sequences in both analyzed databases did not exceed 3.6%.

The results for each construct obtained during PCR were quantified using DNA calibrators and a calibration curve constructed on their basis. Thus, the result was obtained in copies of DNA per PCR sample. The obtained quantitative results were then normalized to the values obtained in ddPCR for the bla gene. To assess the reliability of the differences for most DNA constructs, the experiment was repeated 3–4 times. The exceptions were those constructs for which a significant decrease in amplification efficiency was observed (constructs with three mutations in the probe binding region).

**Figure 4:**
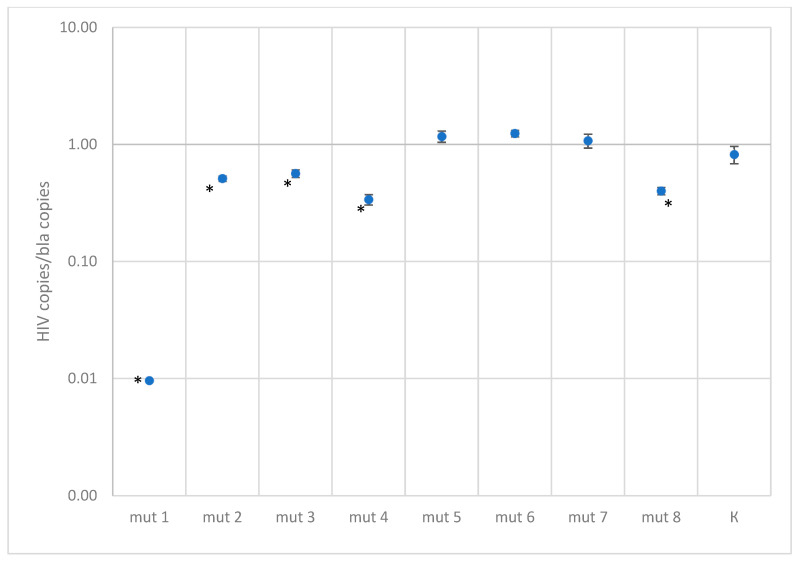
Results of quantitative measurements of constructs with mutations in the primer binding region, normalized to the bla gene (K—control plasmid without mutations. A statistically significant of the difference (*p*-value < 0.001 according to the Mann–Whitney U-test) relative to the control plasmid is shown with asterisk *).

**Figure 5:**
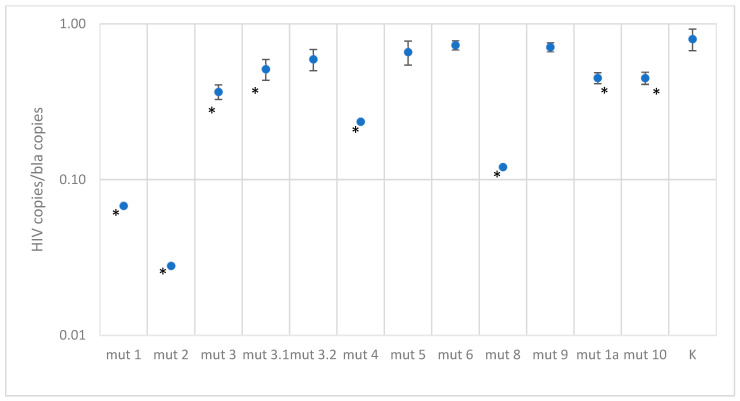
Results of quantitative measurements of constructs with mutations in the probe binding region, normalized to the bla gene (K—control plasmid without mutations. A statistically significant of the difference (*p*-value < 0.001 according to the Mann–Whitney U-test) relative to the control plasmid is shown with asterisk *).

The quantitative results for DNA constructs with mutations are presented in Figure 4 and Figure 5 and in the supplementary materials (Table S1).

Plasmids with single mutations showed quantitative results similar to the control, except for mut3-1, where a two-fold decrease in concentration was observed. For constructs with two mutations in the probe binding region, a slight decrease in quantitative values was observed, by a factor of 1.5–2.7, depending on the construct. As expected, constructs with three mutations at the probe binding site are poorly detected; results are underestimated by a factor of four or more. However, it is worth noting that in the Los Alamos database, three mutations occur simultaneously with a frequency of 1.5%, and among Russian isolates, 0.3%.

In the case of testing plasmids containing mutations in the primer binding sites, the situation turned out to be more optimistic. Most constructs were effectively detected using primers. The only exception was one construct, mut 1, which had the closest location of substitutions to the 3′ end of the primer and to each other, forming two consecutive TG mismatches and another one across the nucleotide—the quantitative result was underestimated by a factor of 100. This sequence variant in the Los Alamos database is represented by the only isolate belonging to group O. A reduced amplification efficiency (2.5–3 times) was also recorded when testing the mut4 construct, which has five substitutions, two of which are located one after another in the middle of the forward primer binding site, and mut8 construct, which has four substitutions in the reverse primer region. Sequences similar to these two constructs were not found in the analyzed databases.

Despite the statistically significant difference between the control plasmid and the mut2 and mut3 constructs, the quantitative result was underestimated by less than two times.

### 3.4. Selection of a Conserved Target for Discordant Sequences

**Figure 6:**
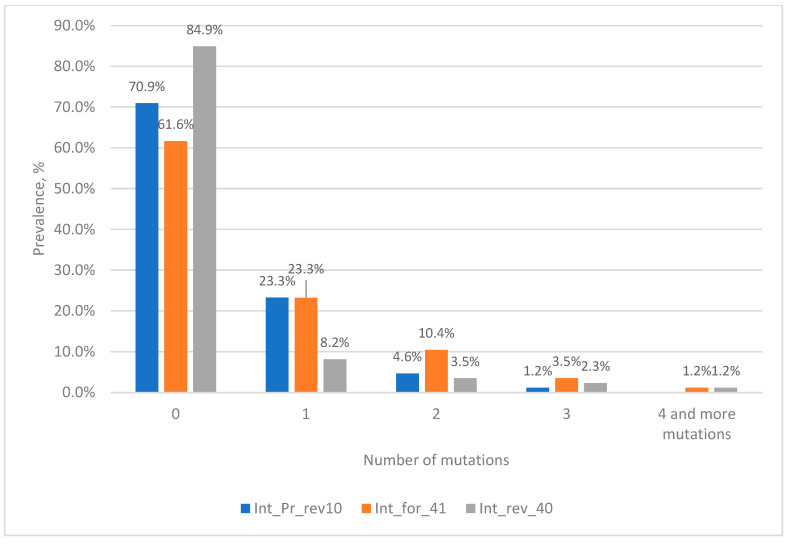
Prevalence of HIV-1 strains with mutations in primers and probe binding sites.

To effectively identify discordant samples, we attempted to select an additional target for real-time PCR. For this purpose, we aligned 86 nucleotide sequences with three or more mutations in the probe binding region and conducted a search for conserved regions to select the primers and probe. The most conserved region was found to be the same region of integrase in the immediate vicinity of the first target. Within this region, we selected the primers int_for_41 and int_rev_40 and the probe int_Pr_rev10 (Figure 6). The analysis of the frequency of mutations in the region of the primer and probe showed that in the probe region, only one sequence has three mutations, which belongs to HIV group N, and in the primers region, there is one sequence each with four mutations.

## 4. Discussion

Due to the high variability of HIV-1, finding a single highly conserved target sequence for the effective detection of HIV-1 RNA via Real-Time The peal-Time PCR is challenging. In recent years, a number of experts have expressed the idea of the need to select at least two targets for HIV-1 amplification, thereby ensuring oneself in the event of mutations in one of the targets.

In our work, we have shown that the task of choosing primers for the effective amplification of most HIV-1 variants is quite feasible. The amplification conditions were selected so that 2–3 mismatches in the primer annealing region did not lead to a decrease in amplification efficiency. A more complex situation is the choice of a TaqMan probe, since its selection requires more stringent requirements, in particular, short length, high melting temperature, and a minimum of mutations. Our findings demonstrate that, in most cases, 1–2 mutations at the probe binding site do not significantly impact detection efficiency; therefore, according to our data, such a probe will detect approximately 98.5% of all viruses from the analyzed databases with similar efficiency. The problem remains with the remaining 1.5% of viruses, in the sequence of which there are three or more mutations at the probe binding site and for which a significant decrease in PCR efficiency has been shown. In this case, the most obvious tactic is to select a second target. On the one hand, this is an insignificant percentage of strains that will be under-detected throughout the world, while in one specific region, for example, in Russia, this percentage is much lower (0.3%). On the other hand, achieving 100% detection using one target is unlikely, since it is impossible to predict the occurrence of certain mutations.

Developers of HIV-1 viral load kits predominantly target the polymerase gene, particularly its highly conservative integrase gene (examples include Abbott m2000 sp/rt RealTime HIV-1, Alinity m HIV-1 assay, Siemens Versant^®^ kPCR HIV-1, and Hologic Panther^®^ Aptima HIV-1). Only the Roche cobas^®^ AmpliPrep/cobas TaqMan (CAP/CTM) HIV-1 v2.0 kits, following an older tradition, utilize a target in the gag region. As this region is not highly conservative, Roche Molecular Systems was among the first to introduce a second target into their kits, specifically in the LTR region. Subsequently, other manufacturers also started incorporating a second target, predominantly in the LTR region (for example, Alinity m HIV-1 assay), considering it to be sufficiently conservative. This approach has notably impacted Russian kit developers. However, in our opinion, this approach has a significant drawback: the number of LTR nucleotide sequences in international databases is much smaller than, for example, in the polymerase region. Thus, in the Los Alamos database, there are 6802 “web complete sequences” in the pol region and 1305 in LTR. If you search the NCBI database using the keywords “HIV-1 and pol gene”, more than 580,000 sequences are downloaded; a similar search for the LTR gene yields about 10,000 sequences. Evaluating the conservation level of this target proves to be quite challenging. Moreover, it is now becoming clear that Roche Molecular Systems was forced to take this step, since a number of publications have accumulated showing that kits with a single target in the gag gene under-detect a number of strains [10]. The addition of a second target improved the analytical performance of the kit and increased the efficiency of identifying discordant samples [11]. However, it did not completely resolve the issue. Thus, when comparing two kits, COBAS TaqMan HIV-1 Test v2.0 (uses two targets—gag and LTR) and Abbott m2000 Real-Time HIV-1 assay (uses one target—integrase), one sample related to HIV subtype C was identified. This sample gave a lower viral load value of more than 1 lg in the COBAS TaqMan HIV-1 Test v2.0, as it had several mismatches in both targets [12]. In contrast, the Abbott m2000 Real-Time HIV-1 assay kit, containing only one target, showed higher stability in identifying different subtypes of HIV-1.

Due to the high variability of HIV-1, the need to select a second target for PCR diagnostics is obvious, but this requires a more thorough and meaningful approach aimed at identifying those viruses that are not detected with the first target. We used one of the approaches in our work: we made an alignment for discordant sequences, found conserved regions only for them, and then estimated how broadly the selected target would cover the remaining sequences. We aligned sequences (86 pieces) that were discordant at the probe binding site (three or more mutations). It turned out that, in general, it is possible to find conserved regions for most sequences (with the exception of one sequence from group N with three mutations in the probe binding region and one sequence from group O with four mutations in the forward primer binding region). The most conserved regions are located approximately in the same region of the integrase gene as the first target, for which we were able to select primers and a probe and demonstrate their effectiveness on plasmids with mutations at the binding site of the first probe. In this case, it is even difficult to talk about the second target. However, in our opinion, this is a more meaningful approach than choosing a target in the LTR region. In particular, of the 86 sequences, only 58 had full-genome sequences and only 20 had full-length LTR fragments. However, we were able to make an alignment for 54 sequences in the LTR region, but no conserved regions for primer and probe were found.

More generally, the problem of identifying all possible HIV-1 strains could be solved through choosing two maximally conservative targets independently of each other. In this case, it is desirable that two targets be comparable in terms of sample size. Next, it will be necessary to compare the discordant sequences from the two regions to ensure that one primer pair effectively detects the discordant sequences from the second target. The task is not trivial, but with modern bioinformatics capabilities, it is feasible.

## 5. Conclusions

Thus, in this work, we were able to select a conservative target, select primers and a probe for real-time PCR in it, and prove their high efficiency for identifying the majority of HIV-1 strains (98.5% of the analyzed sequences). And for the remaining discordant samples, we were able to select an additional pair of primers and probe. Obviously, other approaches to selecting a second target can be used.

## Figures and Tables

**Table 1:** DNA-constructs with mutations in the oligonucleotides binding sites.

Construct Name	Binding Sites	Russian Strains (NCBI)				International Strains (Los Alamos)			
		%	Claster	Predominant Genotype		%	Claster	Predominant Genotype	
DNA constructs with mutations in the TaqMan probe binding site									
Mut 5	gggattggggg**a**tacagtgcaggg	2.41%	2	B	48%	9.8%	1	B	47%
Mut 3-2	gggattggggggtacagtgcagg**a**	2.16%	3	B	50%	4.41%	2	B	37.9%
Mut 3-1	gggattggggggtaca**c**tgcaggg	1.25%	4	A6/B	40%/40%	0.87%	6	B	43.4%
Mut 6	gggattggggggtac**t**gtgcaggg	0.58%	6	B	100%	0.22%	19	B	75%
Mut 9	gggattggggg**a**tacagtgcagg**a**	0.42%	11	B	80%	0.63%	8	B	60.5%
Mut 3	gggattggggggtaca**c**tgcagg**a**	0	ND	ND	ND	0.68%	7	O	90%
Mut 1a	gggattggggact**ac**agtgcaggg	0.17%	17	A6/CRF63_02A1	50%/50%	0.33%	10	B	47.4%
Mut 10	gggattggggg**a**ta**t**agtgcaggg	0	ND	ND	ND	0.24%	16	B	46%
Mut 8	gggattgggg**aa**tacagtgcagg**a**	0.25%	14	B	100%	0.03%	49	B	100%
Mut 1	gggattgggg**ac**tac**t**gtgcaggg	0	ND	ND	ND	0.28%	12	B/01_AE	37.5%/37.5%
Mut 2	gggattgggg**ac**ta**t**agtgcaggg	0.08% *	40	A1	100%	0.27%	14	B	33%
Mut 4	gggattgggg**acc**acagtgcaggg	0	ND	ND	ND	0.02% *	133	75_BF1	100%
DNA constructs with mutations in the primers binding sites									
Primer Int for	cagcagtacaaatggcagtattcatycaca								
Mut 1	cagcagtacaaatggcagtatt**tg**t**g**caca	0	ND	ND	ND	0.02% *	209	O	100%
Mut 2	cagcagtacaaatggcagt**c**tt**tg**tccaca	0	ND	ND	ND	0.06%	43	O	100%
Mut 3	cagcagtac**t**aatggcagtat**a**catcca**t**a	0	ND	ND	ND	0.02% *	104	B	100%
Mut 4	ca**a**cagt**c**caaat**aa**ca**a**tattcatccaca	0	ND	ND	ND	0	ND	ND	ND
Mut 5	cagcagt**g**caaatggc**g**gt**t**ttcatccaca	0	ND	ND	ND	0.08%	33	N	100%
Primer Int rev	cctgtattacyactgccccttcacctttcca								
Mut 6	c**t**tg**g**attaccactgccccttc**t**cctttcca	0	ND	ND	ND	0.16%	20	N	100%
Mut 7	c**t**tgtat**g**actactgc**t**cc**c**tcacctttcca	0	ND	ND	ND	0.65%	8	O	89.7%
Mut 8	c**t**tgtat**g**actactgcccctt**t**accttt**t**ca	0	ND	ND	ND	0	ND	ND	ND

* one sequence in the data base, ND—no data, introduced mutations are indicated in bold.

## Data Availability

Data supporting reported results can be requested from the authors.

## Supplementary Materials

The following supporting information can be downloaded at: https://www.mdpi.com/article/10.3390/microorganisms11122838/s1, Figure S1: Los Alamos Database. Region of the HIV-1 genome corresponding to the TaqMan probe; Figure S2: NCBI Database (Russian isolates). Region of the HIV-1 genome corresponding to the TaqMan probe; Figure S3: Los Alamos Database. Region of the HIV-1 genome corresponding to the forward primer; Figure S4: NCBI Database (Russian isolates). Region of the HIV-1 genome corresponding to the forward primer; Figure S5: Los Alamos Database. Region of the HIV-1 genome corresponding to the reverse primer; Figure S6: NCBI Database (Russian isolates). Region of the HIV-1 genome corresponding to the reverse primer; Table S1: Quantitative results obtained in Real-Time PCR and digital PCR for DNA constructs.

## References

[1] Maksimenko L.V., Totmenin A.V., Gashnikova M.P., Astakhova E.M., Skudarnov S.E., Ostapova T.S., Yaschenko S.V., Meshkov I.O., Bocharov E.F., Maksyutov R. (2020). Genetic Diversity of HIV-1 in Krasnoyarsk Krai: Area with High Levels of HIV-1 Recombination in Russia. BioMed Res. Int..

[2] Beloukas A., Psarris A., Giannelou P., Kostaki E., Hatzakis A., Paraskevis D. (2016). Molecular epidemiology of HIV-1 infection in Europe: An overview. Infect. Genet. Evol..

[3] He X., Xing H., Ruan Y., Hong K., Cheng C., Hu Y., Xin R., Wei J., Feng Y., Hsi J.H. (2012). A comprehensive mapping of HIV-1 genotypes in various risk groups and regions across China based on a nationwide molecular epidemiologic survey. PLoS ONE.

[4] GenBank. https://www.ncbi.nlm.nih.gov/genbank.

[5] HIV Sequence Database. https://www.hiv.lanl.gov/content/sequence/NEWALIGN/align.html.

[6] Katoh K., Misawa K., Kuma K.I., Miyata T. (2002). MAFFT: A novel method for rapid multiple sequence alignment based on fast Fourier transform. Nucleic Acids Res..

[7] Fu L., Niu B., Zhu Z., Wu S., Li W. (2012). CD-HIT: Accelerated for clustering the next-generation sequencing data. Bioinformatics.

[8] Li G., Piampongsant S., Faria N.R., Voet A., Pineda-Peña A.-C., Khouri R., Lemey P., Vandamme A.-M., Theys K. (2015). An integrated map of HIV genome-wide variation from a population perspective. Retrovirology.

[9] HIV Sequence Compendium 2021. https://www.hiv.lanl.gov/content/sequence/HIV/COMPENDIUM/2021compendium.html.

[10] Korn K., Weissbrich B., Henke-Gendo C., Heim A., Jauer C.M., Taylor N., Eberle J. (2009). Single-point mutations causing more than 100-fold underestimation of human immunodeficiency virus type 1 (HIV-1) load with the Cobas TaqMan HIV-1 real-time PCR assay. J. Clin. Microbiol..

[11] Damond F., Avettand-Fenoel V., Collin G., Roquebert B., Plantier J.C., Ganon A., Sizmann D., Babiel R., Glaubitz J., Chaix M.L. (2010). Evaluation of an upgraded version of the Roche Cobas AmpliPrep/Cobas TaqMan HIV-1 test for HIV-1 load quantification. J. Clin. Microbiol..

[12] Karasi J., Dziezuk F., Quennery L., Förster S., Reischl U., Colucci G., Schoener D., Seguin-Devaux C., Schmit J. (2011). High correlation between the Roche COBAS® AmpliPrep/COBAS® TaqMan® HIV-1, v2.0 and the Abbott m2000 RealTime HIV-1 assays for quantification of viral load in HIV-1 B and non-B subtypes. J. Clin. Virol..

