# Supplementary material for: Analysis of the Frequency of Mutations at Diagnostic Oligonucleotide Sites and Their Impact on the Efficiency of PCR for HIV-1
Source: Microorganisms. 2023 Nov 22;11(12):2838. doi: 10.3390/microorganisms11122838 (PMC10746099; doi:10.3390/microorganisms11122838)
Supplement: Supplementary file 1 [file microorganisms-11-02838-s001.zip › microorganisms-2705335-supplementary.pdf]

Supplementary materials

Name	GGGATTGGGGGGTACAGTGCAGGG	Pct	Count
cluster 0	....	70.03%	4430
cluster 1	...........A............	9.8%	620
cluster 2	.......................A	4.41%	279
cluster 3	..........A.............	2.97%	188
cluster 4	..............T.........	1.77%	112
cluster 5	........A...............	1.5%	95
cluster 6	................C.......	0.87%	55
cluster 7	................C......A	0.68%	43
cluster 8	...........A...........A	0.63%	40
cluster 9	.........A..............	0.6%	38
cluster 10	..........AC............	0.33%	21
cluster 11	...........T............	0.28%	18
cluster 12	..........AC...T........	0.28%	18
cluster 13	.............G..........	0.27%	17
cluster 14	..........AC..T.........	0.27%	17
cluster 15	..................C.....	0.25%	16
cluster 16	...........A..T.........	0.24%	15
cluster 17	.......................R	0.24%	15
cluster 18	...........R............	0.24%	15
cluster 19	...............T........	0.22%	14
cluster 20	..............T........A	0.22%	14
cluster 21	..........A............A	0.17%	11
cluster 22	..............Y.........	0.14%	9
cluster 23	..........A...T.........	0.13%	8
cluster 24	........R...............	0.11%	7
cluster 25	..........AA............	0.09%	6
cluster 26	....................T...	0.09%	6
cluster 27	....................G...	0.09%	6
cluster 28	.................C......	0.08%	5
cluster 29	..A.....................	0.08%	5
cluster 30	...........A....C.......	0.08%	5
cluster 31	.......C................	0.08%	5
cluster 32	........A..............A	0.06%	4
cluster 33	..........AT..T.........	0.06%	4
cluster 34	..........AT...T........	0.06%	4
cluster 35	..........R.............	0.05%	3
cluster 36	...........C............	0.05%	3
cluster 37	..........AC..T.C.......	0.05%	3
cluster 38	...C....................	0.05%	3
cluster 39	...........R...........A	0.05%	3
cluster 40	...........A......C.....	0.05%	3
cluster 41	........A.....T.........	0.05%	3
cluster 42	C	0.03%	2
cluster 43	...........T...........A	0.03%	2
cluster 44	..........AA....C.......	0.03%	2
cluster 45	............C...........	0.03%	2
cluster 46	.........A.............A	0.03%	2
cluster 47	........A.......C.......	0.03%	2
cluster 48	.........A.A............	0.03%	2
cluster 49	..........AA...........A	0.03%	2
cluster 50	...........A...........R	0.03%	2
cluster 51	........T...............	0.03%	2

Figure S1 - Los Alamos Database. Region of the HIV-1 genome corresponding to the TaqMan probe.

Clusters with frequently occurring single mutations are highlighted in a frame.

Name	GGGATTGGGGGGTACAGTGCAGGG	Pct	Count
cluster 0	....	81.03%	974
cluster 1	..........A.............	6.07%	73
cluster 2	...........A............	2.41%	29
cluster 3	.......................A	2.16%	26
cluster 4	................C.......	1.25%	15
cluster 5	..............T.........	0.75%	9
cluster 6	...............T........	0.58%	7
cluster 7	........A...............	0.42%	5
cluster 8	..........R.............	0.42%	5
cluster 9	.......................R	0.42%	5
cluster 10	..............Y.........	0.42%	5
cluster 11	...........A...........A	0.42%	5
cluster 12	.................C......	0.33%	4
cluster 13	...........R............	0.33%	4
cluster 14	..........AA...........A	0.25%	3
cluster 15	.................Y......	0.17%	2
cluster 16	..........A............A	0.17%	2
cluster 17	..........AC............	0.17%	2
cluster 18	........R...............	0.17%	2
cluster 19	................A.......	0.08%	1
cluster 20	.....G...........G......	0.08%	1
cluster 21	....................T...	0.08%	1
cluster 22	.................W......	0.08%	1
cluster 23	.......................N	0.08%	1
cluster 24	..........A...T.........	0.08%	1
cluster 25	.....G..................	0.08%	1
cluster 26	..................C.....	0.08%	1
cluster 27	...........R...........R	0.08%	1
cluster 28	..........C...T.........	0.08%	1
cluster 29	.RK.......WA.R....S....A	0.08%	1
cluster 30	..........R.....S.......	0.08%	1
cluster 31	..............G.........	0.08%	1
cluster 32	.A......................	0.08%	1
cluster 33	....................G...	0.08%	1
cluster 34	R.........A.............	0.08%	1
cluster 35	...........R....C.......	0.08%	1
cluster 36	..R.....................	0.08%	1
cluster 37	.......K................	0.08%	1
cluster 38	........A...W...........	0.08%	1
cluster 39	......T.TA.AG.A.TG.A.AA.	0.08%	1
cluster 40	..........AC..T.........	0.08%	1
cluster 41	.......A................	0.08%	1
cluster 42	..........A.....C.......	0.08%	1
cluster 43	...........A..T.........	0.08%	1

Figure S2 - NCBI Database (Russian isolates). Region of the HIV-1 genome corresponding to the TaqMan probe.

Clusters with frequently occurring single mutations are highlighted in a frame.

3' → 5'

Name	CAGCAGTACAAATGGCAGTATTCATYCACA	Pct(%)	Count
cluster 0	....	73.07	4624
cluster 1	..........G...................	6.62	419
cluster 2	.......G......................	3.05	193
cluster 3	...................G..........	1.96	124
cluster 4	....................C.........	1.6	101
cluster 5	......................T.......	1.44	91
cluster 6	......................TG......	1.02	64
cluster 7	.........................A....	0.82	52
cluster 8	...................C..........	0.68	43
cluster 9	................G.............	0.47	30
cluster 10	.......................G......	0.41	26
cluster 11	...................T..........	0.4	25
cluster 12	.......C......................	0.34	22
cluster 13	.....T........................	0.34	21
cluster 14	....T.........................	0.32	20
cluster 15	.G............................	0.31	20
cluster 16	....G.........................	0.3	19
cluster 17	..........G........G..........	0.29	18
cluster 18	.........T....................	0.26	17
cluster 19	............................T.	0.26	17
cluster 20	.....C........................	0.22	14
cluster 21	.......G..G...................	0.19	12
cluster 22	..........G..............A....	0.17	11
cluster 23	.T............................	0.17	11
cluster 24	..........R...................	0.15	10
cluster 25	...................GC.........	0.13	8
cluster 26	...................R..........	0.11	7
cluster 27	......................Y.......	0.11	7
cluster 28	.......R......................	0.11	7
cluster 29	.......T......................	0.11	7
cluster 30	.......G...........G..........	0.11	7
cluster 31	..........G.........C.........	0.1	6
cluster 32	................C.............	0.08	5
cluster 33	.......G........G..T..........	0.08	5
cluster 34	.....................A........	0.08	5
cluster 35	................R.............	0.08	5
cluster 36	.................A............	0.08	5
cluster 37	.R............................	0.08	5
cluster 38	................T.............	0.08	5
cluster 39	....C.........................	0.07	4
cluster 40	......................A.......	0.07	4
cluster 41	.C............................	0.07	4
cluster 42	....................Y.........	0.07	4
cluster 43	...................C..TG......	0.06	4
cluster 44	.............................G	0.05	3
cluster 45	...........................G..	0.05	3
cluster 46	...........................T..	0.05	3
cluster 47	.G........G...................	0.05	3
cluster 48	..................C...........	0.05	3
cluster 49	.......G...........C..........	0.05	3
cluster 50	.......G............C.........	0.05	3
cluster 51	.....A........................	0.05	3
cluster 52	.......M......................	0.05	3
cluster 53	...........................W..	0.05	3
cluster 54	..A...........................	0.05	3
cluster 55	..........G.....G.............	0.05	3
cluster 56	..........G............G......	0.05	3
cluster 57	..........G...........T.......	0.05	3
cluster 58	.......T..G...................	0.05	3
cluster 59	..........G...........TG......	0.05	3
cluster 60	.......T..............TG......	0.05	3
cluster 61	.W............................	0.04	2

Figure S3 - Los Alamos Database. Region of the HIV-1 genome corresponding to the forward primer.

3` → 5`

Name	CAGCAGTACAAATGGCAGTATTCATYCACA	Pct	Count
cluster 0	....	76.36	921
cluster 1	..........G...................	6.97	84
cluster 2	................G.............	1.74	21
cluster 3	......................T.......	1.32	16
cluster 4	.......G......................	1.08	13
cluster 5	....G.........................	1.0	12
cluster 6	..........R...................	0.99	12
cluster 7	...................G..........	0.83	10
cluster 8	.........................A....	0.83	10
cluster 9	.......R......................	0.83	10
cluster 10	....................C.........	0.75	9
cluster 11	...................R..........	0.5	6
cluster 12	.....C........................	0.41	5
cluster 13	.....T........................	0.33	4
cluster 14	...................C..........	0.33	4
cluster 15	......................Y.......	0.33	4
cluster 16	.G............................	0.25	3
cluster 17	......................TG......	0.25	3
cluster 18	.......W......................	0.17	2
cluster 19	.......C......................	0.17	2
cluster 20	................R.............	0.17	2
cluster 21	.......T........G..C..........	0.17	2
cluster 22	.........T....................	0.17	2
cluster 23	..........G...........T.......	0.17	2
cluster 24	....R.........................	0.17	2
cluster 25	...................T..........	0.17	2
cluster 26	....T.........................	0.17	2
cluster 27	.........................W....	0.17	2
cluster 28	.....T.....TG....T.....T...C..	0.08	1
cluster 29	.........W....................	0.08	1
cluster 30	.W............................	0.08	1
cluster 31	....G..C......................	0.08	1
cluster 32	............................T.	0.08	1
cluster 33	.......................R......	0.08	1
cluster 34	...............G..............	0.08	1
cluster 35	....C.....G...................	0.08	1
cluster 36	.....AC.......................	0.08	1
cluster 37	.R............................	0.08	1
cluster 38	.......R.T....................	0.08	1
cluster 39	..........G..............A....	0.08	1
cluster 40	.T............................	0.08	1
cluster 41	.......MA.....................	0.08	1
cluster 42	.....R........................	0.08	1
cluster 43	.....T................T.......	0.08	1
cluster 44	...........................R..	0.08	1
cluster 45	......W.......................	0.08	1
cluster 46	...................G.....A....	0.08	1
cluster 47	.......R...........M..........	0.08	1
cluster 48	................T.............	0.08	1
cluster 49	Y.............................	0.08	1
cluster 50	...................C..T.......	0.08	1
cluster 51	.......M......................	0.08	1
cluster 52	....G...........G.............	0.08	1
cluster 53	.G..G.........................	0.08	1
cluster 54	................M.............	0.08	1
cluster 55	............................Y.	0.08	1
cluster 56	................G..G..........	0.08	1
cluster 57	................G.........M...	0.08	1
cluster 58	.......R..G...................	0.08	1
cluster 59	....G.....................S...	0.08	1
cluster 60	....M.........................	0.08	1
cluster 61	.......R..R...................	0.08	1

Figure S4 - NCBI Database (Russian isolates). Region of the HIV-1 genome corresponding to the forward primer.

3' ← 5'

Name	TGGAAAGGTGAAGGGGCAGTRGTAATACAGG	Pct (%)	Count
cluster 0	.............................A.	62.13	3929
cluster 1	....	13.36	845
cluster 2	.......................C.....A.	4.36	276
cluster 3	.......................G.....A.	4.13	261
cluster 4	..............A..............A.	2.17	137
cluster 5	.................G...........A.	1.83	116
cluster 6	..........................T..A.	1.61	102
cluster 7	........................C....A.	1.05	66
cluster 8	...........G..A........C.....A.	0.65	41
cluster 9	.......................G.......	0.57	36
cluster 10	.................G.............	0.55	35
cluster 11	.......................T.....A.	0.34	21
cluster 12	..............A................	0.31	20
cluster 13	.............................AC	0.30	19
cluster 14	.......................C.......	0.27	17
cluster 15	..............A........C.....A.	0.19	12
cluster 16	..............A........G.....A.	0.17	11
cluster 17	.............................R.	0.16	10
cluster 18	..........................C..A.	0.16	10
cluster 19	...........G..A..............A.	0.16	10
cluster 20	........A.................C..A.	0.16	10
cluster 21	..........................T....	0.14	9
cluster 22	....................T........A.	0.13	8
cluster 23	.................G.....G.....A.	0.13	8
cluster 24	.......................R.....A.	0.13	8
cluster 25	.................T...........A.	0.13	8
cluster 26	.............................AT	0.12	8
cluster 27	..................A..........A.	0.11	7
cluster 28	..........................C....	0.11	7
cluster 29	........C....................A.	0.11	7
cluster 30	...........................A.A.	0.09	6
cluster 31	...........G.................A.	0.09	6
cluster 32	.................C...........A.	0.09	6
cluster 33	.................G.....C.....A.	0.08	5
cluster 34	....G........................A.	0.08	5
cluster 35	........................C......	0.07	4
cluster 36	........A....................A.	0.07	4
cluster 37	..................A............	0.06	4
cluster 38	C............................A.	0.06	4
cluster 39	........................G....A.	0.06	4
cluster 40	.......................M.....A.	0.06	4
cluster 41	..............R..............A.	0.06	4
cluster 42	....................T..........	0.06	4
cluster 43	.......................G.....AC	0.06	4
cluster 44	...........G..A........T.....A.	0.06	4
cluster 45	.....G.......................A.	0.05	3
cluster 46	....................W........A.	0.05	3
cluster 47	.......................T.......	0.05	3
cluster 48	...............A.............A.	0.05	3
cluster 49	.......................G..T..A.	0.05	3
cluster 50	.................R...........A.	0.05	3
cluster 51	..............T.........C....A.	0.05	3
cluster 52	........................T....A.	0.05	3
cluster 53	..........................M..A.	0.05	3
cluster 54	...........R..A........C.....A.	0.05	3
cluster 55	...........G...........C.....A.	0.05	3
cluster 56	...G.........................A.	0.05	3
cluster 57	........C..............C.....A.	0.04	2
cluster 58	........A......................	0.03	2
cluster 59	..............A..G.............	0.03	2
cluster 60	W............................A.	0.03	2
cluster 61	.....................T.......A.	0.03	2

Figure S5 - Los Alamos Database. Region of the HIV-1 genome corresponding to the reverse primer.

3` ← 5`

Name	TGGAAAGGTGAAGGGGCAGTRGTAATACAGG	Pct	Count
cluster 0	....	76.52	896
cluster 1	.............................A.	12.64	148
cluster 2	.................G.............	3.08	36
cluster 3	.......................G.......	1.28	15
cluster 4	.................G...........A.	1.11	13
cluster 5	.......................G.....A.	0.34	4
cluster 6	........A......................	0.34	4
cluster 7	.................R.............	0.34	4
cluster 8	........C......................	0.26	3
cluster 9	.......................C.......	0.26	3
cluster 10	..............A................	0.26	3
cluster 11	..............R................	0.26	3
cluster 12	.............................R.	0.18	2
cluster 13	....G..........................	0.17	2
cluster 14	..........................T....	0.17	2
cluster 15	........Y......................	0.17	2
cluster 16	.......................C.....A.	0.17	2
cluster 17	..........................T..A.	0.17	2
cluster 18	.......................R.......	0.17	2
cluster 19	.................G.G.........A.	0.09	1
cluster 20	..............A..G...........A.	0.09	1
cluster 21	..A............................	0.09	1
cluster 22	......................G........	0.09	1
cluster 23	.................K.............	0.09	1
cluster 24	..........................M....	0.09	1
cluster 25	.......................M.......	0.09	1
cluster 26	...................K...........	0.09	1
cluster 27	........................C......	0.09	1
cluster 28	.................R.K...........	0.09	1
cluster 29	............................GA.	0.09	1
cluster 30	................T..............	0.09	1
cluster 31	.......................GMK.....	0.09	1
cluster 32	..........................M..A.	0.09	1
cluster 33	..............R..............R.	0.09	1
cluster 34	........................C.G..A.	0.09	1
cluster 35	.................T...........A.	0.09	1
cluster 36	.................R...........A.	0.09	1
cluster 37	.......................R.....A.	0.09	1
cluster 38	....G..................G.......	0.09	1
cluster 39	..........................C....	0.09	1
cluster 40	..R..........................RR	0.09	1
cluster 41	.................C.G...G.......	0.09	1
cluster 42	........A....................AA	0.09	1
cluster 43	.............................AC	0.09	1

Figure S6 - NCBI Database (Russian isolates). Region of the HIV-1 genome corresponding to the reverse primer.

Table S1. Quantitative results obtained in Real-Time PCR and digital PCR for DNA constructs

	ddPCR/Bla		**PCR/HIV**		
Name	Copies/ml	Copies/ml, mean	Copies/ml	Copies/ml, mean	HIV PCR Copies/ml, mean / BLA ddPCR Copies/ml, mean
mut 1	1.96E+04	2.21E+04	296	233	0.01
	2.48E+04		112		
	2.18E+04		291		
mut 2	2.16E+04	2.41E+04	1.34E+04	1.33E+04	0.55
	2.50E+04		1.28E+04		
	2.56E+04		1.37E+04		
			1.23E+04	1.22E+04	0.51
			1.35E+04		
			1.11E+04		
			1.24E+04	1.17E+04	0.49
			1.20E+04		
			1.08E+04		
			1.19E+04	1.22E+04	0.50
			1.20E+04		
			1.26E+04		
mut 3	2.58E+04	2.48E+04	1.27E+04	1.34E+04	0.54
	2.10E+04		1.38E+04		
	2.76E+04		1.36E+04		
			1.53E+04	1.45E+04	0.59
			1.32E+04		
			1.52E+04		
			1.66E+04	1.53E+04	0.62
			1.45E+04		
			1.48E+04		
			1.32E+04	1.30E+04	0.52
			1.48E+04		
			1.13E+04		
mut 4	2.96E+04	2.60E+04	8.56E+03	9.18E+03	0.35
	2.12E+04		1.09E+04		
	2.72E+04		8.32E+03		
			8.15E+03	9.55E+03	0.37
			9.94E+03		
			1.07E+04		
			1.03E+04	8.96E+03	0.34
			8.26E+03		
			8.45E+03		
			8.82E+03	7.50E+03	0.29
			6.95E+03		
			6.90E+03		
mut 5	2.78E+04	2.43E+04	2.54E+04	2.63E+04	1.08
	2.38E+04		2.55E+04		
	2.12E+04		2.82E+04		
			3.54E+04	3.31E+04	1.36
			3.13E+04		
			3.26E+04		
			2.88E+04	2.79E+04	1.15

			2.61E+04		
			2.90E+04		
			2.65E+04	2.65E+04	1.09
			2.47E+04		
			2.85E+04		
mut 6	2.10E+04	2.44E+04	3.54E+04	3.12E+04	1.28
	2.68E+04		2.71E+04		
	2.54E+04		3.17E+04		
			3.48E+04	3.20E+04	1.31
			3.08E+04		
			3.05E+04		
			2.84E+04	3.07E+04	1.26
			3.56E+04		
			2.87E+04		
			2.92E+04	2.75E+04	1.13
			2.52E+04		
			2.83E+04		
mut 7	3.12E+04	2.87E+04	3.35E+04	3.04E+04	1.06
	2.76E+04		2.88E+04		
	2.72E+04		2.90E+04		
			3.49E+04	3.59E+04	1.25
			3.81E+04		
			3.47E+04		
			3.06E+04	3.17E+04	1.11
			2.87E+04		
			3.63E+04		
			2.69E+04	2.57E+04	0.89
			2.74E+04		
			2.30E+04		
mut 8	2.82E+04	2.77E+04	9.53E+03	1.04E+04	0.38
	2.90E+04		1.17E+04		
	2.60E+04		1.02E+04		
			1.10E+04	1.14E+04	0.41
			1.12E+04		
			1.22E+04		
			1.20E+04	1.21E+04	0.44
			1.29E+04		
			1.15E+04		
			1.17E+04	1.05E+04	0.38
			8.26E+03		
			1.21E+04		
K+	2.84E+04	3.11E+04	2.81E+04	2.40E+04	0.77
	3.20E+04		2.36E+04		
	3.30E+04		2.10E+04		
			2.43E+04	3.17E+04	1.02
			4.63E+04		
			2.83E+04		
			1.88E+04	2.50E+04	0.80
			2.08E+04		
			3.98E+04		
			2.24E+04	2.16E+04	0.70

			2.23E+04		
			2.03E+04		
